# The cell biology of late blight disease

**DOI:** 10.1016/j.mib.2016.09.002

**Published:** 2016-12

**Authors:** Stephen C Whisson, Petra C Boevink, Shumei Wang, Paul RJ Birch

**Affiliations:** 1 Cell and Molecular Sciences, James Hutton Institute, Errol Road, Invergowrie, Dundee DD2 5DA, UK; 2 Division of Plant Sciences, University of Dundee, James Hutton Institute, Invergowrie, Dundee DD2 5DA, UK

## Abstract

Late blight, caused by the oomycete *Phytophthora infestans*, is a major global disease of potato and tomato. Cell biology is teaching us much about the developmental stages associated with infection, especially the haustorium, which is a site of intimate interaction and molecular exchange between pathogen and host. Recent observations suggest a role for the plant endocytic cycle in specific recruitment of host proteins to the Extra-Haustorial Membrane, emphasising the unique nature of this membrane compartment. In addition, there has been a strong focus on the activities of RXLR effectors, which are delivered into plant cells to modulate and manipulate host processes. RXLR effectors interact directly with diverse plant proteins at a range of subcellular locations to promote disease.

## Highlights

• The *Phytophthora* haustorium is a major site of secretion during infection.
• The host endocytic cycle contributes to biogenesis of the Extra-Haustorial Membrane.
• RXLR effectors manipulate host processes at diverse subcellular locations.
• They directly manipulate the activity or location of immune proteins.
• They also promote the activity of endogenous negative regulators of immunity.

**Current Opinion in Microbiology** 2016, **34**:127–135

This review comes from a themed issue on **Parasitic and fungal diseases**

Edited by **Gero Steinberg**

For a complete overview see the Issue and the Editorial

Available online 7th October 2016

**http://dx.doi.org/10.1016/j.mib.2016.09.002**

1369-5274/© 2016 The Authors. Published by Elsevier Ltd. This is an open access article under the CC BY license (http://creativecommons.org/licenses/by/4.0/).

## Introduction

Late blight is a devastating disease of potato and tomato. The organism that causes it, *Phytophthora infestans*, is an oomycete; related to diatoms and brown algae. Its discovery in the late 19th century contributed to the establishment of plant pathology as a research discipline. Late blight remains the number one potato disease nearly 150 years later. The global population of *P. infestans* constantly changes, with emergence of aggressive new strains, ensuring that late blight continues to be an ongoing threat to global food security [1, 2].

The development of late blight disease can be better understood through the cell biology of both pathogen and host, and especially the intimate points of contact between them, epitomised by the haustorial interface. Effort has focussed in recent years on the activities of effectors that are delivered inside plant cells; on where they go, what they target and what they alter to facilitate infection. Whilst this review broadly introduces *P. infestans* cell biology, we highlight the molecular interactions and exchanges between pathogen and host that dictate disease or disease resistance, and describe the approaches used to observe these events in close detail.

## The infection cycle

**Figure 1:**
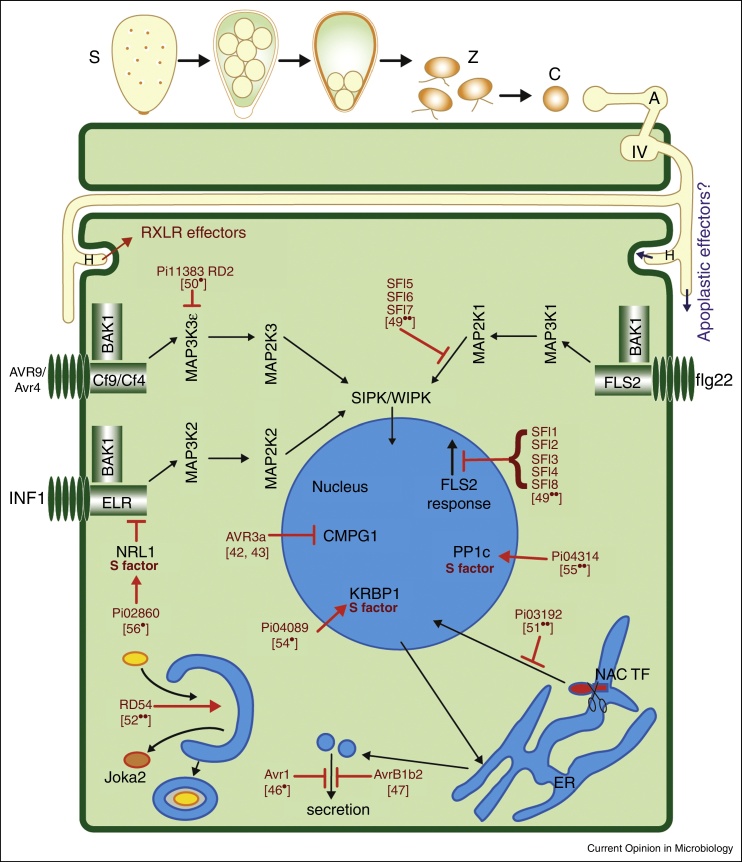
Diagram of the *P. infestans* infection cycle and the many roles effectors play in modulating plant cellular processes. The main dispersal stage is the multinucleate sporangium (S) which either germinates directly or releases zoospores (Z). Zoospores rapidly encyst (C) on a host plant then germinate to form an appressorium-like (A) swelling at the end of the germ tube, under which penetration takes place to form the infection vesicle (IV). From this intercellular hyphae extend and grow between host cells, projecting haustoria (H) into cells. The haustoria are the sites of secretion of the RXLR class of effectors (shown in red) and some of their characterised protein targets and activities are represented in this diagram. Several effectors are attuned to suppress signal transduction pathways emanating from membrane-bound, BRASSINOSTEROID-ASSOCIATED KINASES 1 (BAK1)-dependent receptors such as FLAGELLIN-SENSING 2 (FLS2), and can act redundantly. Some effectors act to inhibit specific immune response factors and pathways, while others promote the activity of negative regulators which can thus be regarded as susceptibility factors (S factors). Several effectors target diverse nuclear-located processes while others target processes involving the endoplasmic reticulum (ER), vesicles in the secretory pathway, the plasma membrane, or autophagosomes.

There are over 120 known species of *Phytophthora* [3] and all are pathogens of plants. They colonise different host tissues, such as roots, tubers, herbaceous stems, woody trunks, foliage, and fruit. *Phytophthora* species develop distinct cellular stages in their infection cycle [4]. Multinucleate sporangia and uninucleate motile zoospores represent the primary dispersal stages. *P. infestans* sporangia formed on aerial plant parts may be blown or splashed to new hosts, where they may either germinate directly, or release zoospores, to initiate infection. Zoospores discard flagella and synthesise a cell wall, forming a cyst. These germinate within hours and may enter host tissue through natural openings such as stomata, or form an appressorium-like swollen germ tube, beneath which penetration of host epidermal cells occurs. Upon host cell penetration, a spherical primary infection vesicle is formed from which hyphae emerge to ramify through plant tissue. *P. infestans* hyphae grow intercellularly, projecting digit-like haustoria into host cells [5, 6, 7, 8]. Haustoria are structures that form an intimate interaction with host cells, removing the plant cell wall but leaving the membrane intact to facilitate molecular exchange between the pathogen and a living plant cell. It is of interest to reveal how these specialised cell types differ from one another and how transition is regulated from one stage to the next: sporangium → zoospore → cyst → germination → appressorium → host penetration and infection vesicle → intercellular hyphal growth → haustorium formation → initiation of sporulation. Knowledge of the differences and similarities between these developmental stages will facilitate novel means to control infection through targeted inhibition of regulatory processes in the pathogen. Infection vesicles, haustoria and intercellular hyphae are of particular interest, as these stages are in close contact with plant cells and this is where the outcome of infection is determined (Figure 1).

## Cell biology of protein localisation in developmental stages

It is challenging to determine the fate or origins of individual pathogen structures, or determine the time-scales over which some developmental events occur. Adoption of the jellyfish green fluorescent protein (GFP) for examining cell biology in a multitude of organisms began in the mid-1990s. Many other fluorescent proteins (FPs) have since been developed, with emission spectra covering all visible light [9]. The expression of FPs relies on the delivery of foreign DNA into an organism to stably or transiently express these transgenes. Stable transformation of *P. infestans* and other *Phytophthora* species has been achieved by a variety of methods [10], with GFP-expressing *P. parasitica* and *P. palmivora* being the first recorded fluorescently-labelled species [11, 12]. Other *Phytophthora* species have since been labelled with GFP or other FPs [13, 14, 15]. Fluorescently-labelled strains of *P. infestans* allow investigation of the life cycle and the dynamics of infection in living host tissue.

In *P. infestans*, proteins have been localised to specific lifecycle stages as FP-fusions. However, there are relatively few studies where the dynamics of the localised proteins have been followed systematically through the entire infection cycle. For example, use of the LifeAct-GFP tag for live actin localisation [16] revealed that actin in *P. infestans in vitro* cultured hyphae exists as long-lived plaques and highly dynamic filaments [17]. Future studies may reveal actin dynamics associated with penetration of the host and haustorium formation.

Similarly, two oomycete-specific G protein coupled receptors with phosphatidylinositol phosphate kinase domains (called GK1 and GK4) were localised to vesicles in germinating *P. infestans* sporangia *in vitro* [18]. Although the two GKs are expressed throughout infection, and GK4 and G protein-mediated signalling are required for full pathogenicity [18, 19], the behaviour of GK1 and GK4 have yet to be investigated during infection.

Additional proteins that have been localised in *P. infestans* include the bZIP transcription factor PITG_11668 in the nucleus [20], Picdc14 in the nucleus and flagella basal body [21], and Argonaute in the cytoplasm [22]. FP fusion markers for subcellular organelles such as golgi, endoplasmic reticulum, peroxisomes, and mitochondria have been expressed in *P. infestans*, but have only been examined in cultured hyphae and have yet to be studied during infection [23].

## The haustorium as a site of molecular exchange with the host

*P. infestans* haustorium formation was first examined by electron microscopy (EM) in potato leaf tissue [24]. This and later studies revealed a complex host–pathogen interface where haustoria are bounded by a pathogen cell wall, and surrounded by the host cell. The ‘space’ between the haustorial wall and the host cell membrane is referred to as the extrahaustorial matrix (EHMx). The EHMx possesses protuberances that interlink with plant cell membrane invaginations, suggesting that *P. infestans* haustoria establish intimate interactions with the host. The haustorium wall is continuous with the hyphae, without the neck or neckband observed in fungal haustoria. Host cell wall appositions (callose) were observed to be generally absent, or formed a collar-like structure near the base of haustoria in susceptible host tissue, while such appositions fully encased haustoria in resistant host tissues [5, 6, 25].

Microbes suppress plant immunity by the secretion of so-called effector proteins that can act either outside (apoplastic effectors) or inside (cytoplasmic effectors) the host cell. *P. infestans* is no exception. The avirulence protein Avr3a (PiAvr3a), a member of the RXLR class of cytoplasmic effectors [26], was fused to monomeric red fluorescent protein (mRFP) and localised to haustoria [7]. A similar mRFP fusion to PiAvr3a, but with the RXLR motif mutated to alanine residues, showed red fluorescence apparently leaking out into the apoplast from around the haustorium. Mutation of the RXLR motif prevented the effector entering plant cells, presumably resulting in its accumulation in the EHMx. One interpretation of the observed leakage of the mutant protein is that the EHMx is not a compartment sealed from the apoplast. This was consistent with observations from earlier EM studies that did not identify a haustorium neckband that would seal off the EHMx as a discrete compartment [5, 6, 25]. Other RXLR effectors, such as PiAvr2 [27], PiAvr4, and PiAvrBlb1 (IPIO) [28] have also been localised to haustoria, implicating this as a site of secretion and delivery of these effectors into host cells (Figure 1). Remarkably, although predicted apoplastic effectors such as the cystatin-like protein PiEPIC1 [29] have been functionally well characterised, the sites at which they are secreted by the pathogen during infection are unknown.

The infection-induced haustorium membrane protein 1 (PiHmp1) protein, predicted to be membrane localised, was also shown to reside in the haustorial membrane [8]. Expression of an *PiHmp1–mRFP* fusion was driven by its native gene promotor, allowing the temporal aspects of protein translation, transport, and localisation to be determined during plant colonisation. PiHmp1 was observed to be initially translated and stored in vesicles in germinating cysts and appressoria, after which it was localised to the haustorium surface. During haustorium development, PiHmp1 first localised to small plaques on the surface of hyphae. These plaques became the sites at which haustoria were formed. The haustoria entered host cells within three hours, and developed into the characteristic hooked digit shape over a 12 h period. Transient silencing of *PiHmp1* prevented infection, demonstrating the importance of haustoria to host colonisation [8].

Although *P. infestans* is the most intensively studied oomycete [7, 8, 27, 28], secreted effectors and additional infection-related proteins from other *Phytophthora* species have been localised during plant infection. In *P. sojae*, the RXLR effector PsAvr1b was localised specifically to haustoria [30^•^]. In the same study, a non-conventionally secreted isochorismate mutase was also shown to accumulate preferentially, but not exclusively, at *P. sojae* haustoria. In *P. parasitica*, a secreted protein disulphide isomerase also localised to haustoria [31]. These, and results from *P. infestans*, suggest that *Phytophthora* haustoria are major sites of protein export during infection, and that both conventional and non-conventional secretion occurs at these structures.

## Biogenesis of the extra-haustorial membrane

A host-derived membrane, the extra-haustorial membrane (EHM) separates the haustorium and the EHMx from the invaded plant cell. Recent studies have shown that the EHM is a distinct membrane compartment; a number of plant plasma membrane (PM)-localised proteins are excluded from the EHM in *P. infestans*-infected cells, whereas others such as remorin REM1.3 and synaptotagmin SYT1 are localised to the EHM. Moreover, significant re-programming is apparent in haustoriated cells, perhaps indicating that the plant endocytic cycle contributes to the biogenesis of the EHM [32^•^]. Using super-resolution confocal microscopy, REM1.3 was shown to co-localise with the effector PiAvrblb2 at discrete EHM domains. By contrast, SYT1 localised to distinct EHM domains. Whereas overexpression of REM1.3 enhanced *P. infestans* leaf colonisation, virus-induced gene silencing (VIGS) of *REM1.3* attenuated infection, suggesting that it acts as a susceptibility (S) factor; a host protein whose activity contributes to disease development [33].

More recently, specific re-routing of plant late endocytic trafficking to the EHM was shown to occur. The Rab7 GTPase RabG3c is a marker of late endosomes and the tonoplast. During *P. infestans* infection, RabG3c was recruited to the EHM, whereas another tonoplast-localised marker, the sucrose transporter SUC4, was not [34^•^]. In addition, the pattern recognition receptor FLS2 was shown to be re-localised to the *P. infestans* EHM specifically following activation by treatment with its ligand flg22 [34^•^]. It will be important not only to better characterise the constituents of the EHM, and whether they are derived from pathogen or host, but also to determine the roles of *P. infestans* effectors in facilitating EHM biogenesis for the benefit of the pathogen, in contrast to the contribution of the plant immune system to dictating EHM composition.

## Secretion and delivery of effectors into host cells

Considerable evidence has accumulated over the past decade to indicate that RXLR effectors function inside host cells. For example, all avirulence proteins detected by cytoplasmic resistance (R) proteins belong to this class of effectors, and a growing body of evidence has provided the cytoplasmic virulence targets of RXLR effectors, revealing their contributions to infection (see below). Nevertheless, there has been controversy about the mechanism by which RXLR effectors are translocated into plant cells from *Phytophthora* haustoria [35, 36, 37, 38, 39]. Delivery of a tagged effector from the pathogen into plant cells has not been directly observed. The closest to direct observation of RXLR effector translocation involved fusion of the RXLR domain of PiAvr3a to the β-glucuronidase (GUS) enzyme to demonstrate effector delivery [7]. However, this assay has not been attempted in other pathosystems with RXLR effectors, and cannot accurately provide subcellular localisation of the translocated effector. PiAvr3a-mRFP fusions were not observed in host cells containing haustoria likely due to dilution within the much larger plant cells, compounded by the low fluorescence intensity of mRFP and the presence of contaminating autofluorescence from tissue damage, making it difficult to be confident about detection of low intensity red signal [7]. In this regard, a labelled effector known to be targeted to a specific host cell organelle, such as the host cell nucleus, may yield the best likelihood of success due to specific accumulation at a discrete site in plant cells. Direct visualisation of an RXLR effector that has been translocated from a *Phytophthora* species into a plant cell would provide unambiguous evidence for effector delivery, and provide greater opportunity for *in vivo* analyses of effector translocation.

Another class of oomycete effectors, termed crinkling and necrosis (CRN), are proposed to act inside host plant cells during infection. Again, evidence of their translocation comes from expression in *P. capsici* or *P. sojae* of N-terminal CRN fusions to RXLR avirulence effector domains, and subsequent detection of cell death in plants expressing the cognate resistance protein [40, 41•]. No CRN effector has been fluorescently tagged in *Phytophthora* species and localised during infection of plants. Further work will hopefully demonstrate by direct observation whether this family of proteins are translocated into plant cells by *Phytophthora* species during infection.

## Sites of action, targets and recognition of RXLR effectors inside host cells

In reviewing the cell biology of late blight disease, it is important to consider the sites of activity of RXLR effectors inside host cells and the identification of their host protein targets. This has been a major research focus in recent years. When delivered into the plant, RXLR effectors are predicted to traffic to a range of subcellular localisations and target diverse host proteins and processes to promote disease. Studies of *P. infestans* effector localisation and function inside plant cells has been greatly facilitated by transient *Agrobacterium tumefaciens*-mediated expression in *Nicotiana benthamiana*, allowing over expression of proteins, or their reduction by virus-induced gene silencing (VIGS). In addition, *P. infestans* can complete its infection cycle in *N. benthamiana*, and this plant thus acts as a model host for cell biology studies of late blight disease.

PiAvr3a was the first RXLR effector from *P. infestans* to be studied in detail. Expressed transiently in *N. benthamiana* as an N-terminal FP fusion protein (CFP-PiAvr3a), lacking the secretion signal peptide, the effector is generally nucleo-cytoplasmic in host cells and retains its ability to suppress pathogen-associated molecular pattern (PAMP)-triggered immunity (PTI) activated by perception of the *P. infestans* Infestin1 (INF1) protein. It does so by interaction *in planta* with the ubiquitin E3 ligase CMPG1, stabilising it and thus preventing its normal activity in promoting INF1-mediated cell death (Figure 1) [42, 43]. Importantly, fusion of CFP to the N-terminus of PiAvr3a, in place of the signal peptide, does not inhibit effector activity, allowing this to be directly correlated with subcellular localisation. The PiAvr3a^KI^ form of the effector is recognised by the potato R3a protein. However, CMPG1 is not required for this recognition, indicating that it is not a ‘guardee’ monitored by R3a [42, 43]. The R3a protein, expressed as an N-terminal FP fusion, is generally cytoplasmic, similar to PiAvr3a. However, when co-expressed in *N. benthamiana* with the recognised PiAVR3a^KI^ form, both R3a and the effector are re-localised to late endosomes [44]. Treatment with inhibitors of the endocytic cycle, such as brefeldin A or wortmannin, attenuated both re-localisation of R3a and PiAvr3a^KI^ to late endosomes and the R3a-mediated hypersensitive response (HR). Thus, effector recognition and consequent HR signalling by R3a require its re-localisation to vesicles in the endocytic pathway [44].

GFP-PiAvr1 has also recently been reported to be nucleo-cytoplasmic when expressed inside *N. benthamiana* cells [45^•^]. PiAvr1 associates with the exocyst subunit Sec5 in yeast-2-hybrid experiments and *in planta*. Bimolecular fluorescence complementation (BiFC) between PiAvr1 and Sec5 indicate that they are in close proximity at mobile vesicles in the host cell (Figure 1) [46^•^]. The exocyst complex is involved in both secretion and endocytosis, and virus-induced gene silencing (VIGS) of *Sec5* resulted in reduced callose deposition and pathogenesis-related 1 (PR1) secretion, and increased leaf colonisation by *P. infestans* [46^•^]. PiAvr1 is recognised by the potato R1 resistance protein. By contrast to the detection of PiAvr3a by R3a, recognition of PiAvr1 by the potato R1 protein occurred only when both proteins were present in the nucleus. Addition of a nuclear export signal (NES) to either R1 or PiAvr1 prevented R1-mediated HR [45^•^]. It has not yet been determined whether R1-mediated HR is dependent on the interaction between PiAvr1 and Sec5.

By contrast to PiAvr3a and PiAvr1, RXLR effectors PiAvrblb2 [47] and PiAvr2 [48] are partially associated with the host plasma membrane (PM) when expressed inside *N. benthamiana* cells, and hyper-accumulate around the sites of haustorium formation during infection. PiAvrblb2 associates *in planta* with vesicles containing a defence-associated protease C14, preventing its secretion into the apoplast (Figure 1) [47]. PiAvr2, however, interacts in yeast-2-hybrid and *in planta* with the putative phosphatase BSL1, which is predicted to be involved in brassinosteroid signal transduction, and thus a positive regulator of growth and development [48]. As yet, the consequence to virulence of PiAvr2 interaction with BSL1 is unknown. However, recognition of PiAvr2 by the potato R2 resistance protein is dependent on BSL1 interaction, suggesting that R2 monitors non-self-modification of BSL1 structure or activity, in line with the Guard Hypothesis [48].

Expression of 35 candidate *P. infestans* RXLR effectors in tomato protoplasts revealed eight that suppressed PTI activated by the bacterial PAMP flg22 (Figure 1) [49^••^], indicating that there is functional redundancy in the effector repertoire. This medium-throughput screen involved flg22-mediated activation of the *FRK1* promoter fused to the reporter luciferase. Whilst *P. infestans* lacks the flg22 peptide that activates the host receptor FLAGELLIN-SENSING 2 (FLS2), the signalling pathway activated by this PAMP is generic, and was suppressed by eight *P. infestans* effectors. This indicates that the pathway is activated by perception of an as yet unknown oomycete PAMP. Of the eight RXLR effectors that attenuated flg22-mediated *pFRK1*-luciferase induction, three also suppressed MAP-kinase activation, indicating that they act upstream of this event. These three effectors showed varying levels of association with the host PM, perhaps indicating that they act on receptor complexes at the plant cell surface. By contrast, one of the effectors, SFI1, that suppressed *pFRK1*-luciferase expression but not MAPK activation, localised to the host nucleus and nucleolus. Addition of an N-terminal myristoylation signal to this effector to make Myr-GFP-SFI1 resulted in its exclusion from the nucleus and accumulation at the host PM [49^••^]. This misdirected form of SFI1 no longer suppressed flg22-induced *pFRK1*-luciferase expression, indicating that the host nucleus/nucleolus was the likely site of effector activity.

RXLR effector PexRD2, expressed in *N. benthamiana* cells, interacts in the cytoplasm with MAP3Kɛ, a positive regulator of cell death that relays signals to promote cell death following perception of *Cladosporium fulvum* CfAvr4 by the tomato Cf4 resistance receptor (Figure 1) [50^•^]. The effector interacts directly with the kinase domain, suppressing kinase activity, but does not interact with the closely related MAP3Kα. Moreover, PexRD2 failed to suppress MAP kinase activation upon flg22 treatment, demonstrating the exquisitely targeted nature of PexRD2 to a specific immune protein and pathway.

The *P. infestans* RXLR effector Pi03192 localised to the endoplasmic reticulum (ER) when expressed in *N. benthamiana* cells. In a yeast-2-hybrid screen, Pi03192 interacted with two potato NAC transcription factors (TFs), NTP1 and NTP2, each of which also localised to the host ER [51^•^]. Upon PAMP treatment, NTP1 and NTP2 were released from the ER and accumulated in the host nucleus. Silencing of *NTP1* and *NTP2* enhanced susceptibility to *P. infestans*, indicating that these TFs are likely to be positive regulators of immunity. Effector Pi03192 prevented re-localisation of the NAC TFs from the ER into the host nucleus (Figure 1), thus providing a simple mode-of-action to promote disease progression that was determined using cell biology [51^•^].

More recently, *P. infestans* RXLR effector PexRD54 was shown to possess an ATG8 interacting motif and to associate *in planta* with the autophagy regulator ATG8CL, stimulating the formation of autophagosomes [52^••^]. Remarkably, PexRD54 acts to promote selective autophagy, excluding the autophagy cargo receptor joka2, a positive regulator of immunity, from ATG8CL complexes (Figure 1). Further work will demonstrate whether the effector, in turn, promotes the formation of autophagosomes with cargo of benefit to the pathogen, perhaps redistributing cellular resources to the haustorial interface as a source of nutrition.

The characterised *P. infestans* RXLR effectors referred to above generally target host proteins that are positive regulators of immunity to inhibit or disrupt their contribution to defence. Given that PexRD54 promotes autophagy, the story of its role in infection may extend beyond the observed exclusion of joka2 from autophagosomes [52^••^]. Recently, *P. infestans* RXLR effectors have also been shown to target host proteins whose activity can play a positive role in promoting disease; so-called susceptibility (S) factors [53]. Effectors Pi04089 [54^•^] and Pi04314 [55^••^] localise in the nucleus and nucleolus when expressed transiently in *N. benthamiana*, where they enhance *P. infestans* colonisation. This promotion of colonisation is attenuated when they are redirected away from the nucleus; Pi04089 by addition of an NES, and Pi04314 by addition of a myristoylation signal. By contrast, RXLR effector Pi02860 enhances *P. infestans* colonisation and suppresses INF1-triggered cell death when excluded from the nucleus, indicating that the nucleus is not the site of its activity in plant cells [56^•^].

Pi04089 interacts with a predicted K-homology RNA binding protein, KRBP1, which localises to nuclear speckles (Figure 1). Co-expression of KRBP1 with Pi04089 indicates that they interact at these speckles, and that the effector increases the abundance of KRBP1. Remarkably, overexpression of KRBP1 enhances *P. infestans* infection; a key criterion in defining it as an S factor [54^•^]. Pi04314 interacts in yeast and *in planta* with three isoforms of protein phosphatase 1 catalytic (PP1c) subunits (Figure 1). The effector promotes re-localisation of the PP1c isoforms from the nucleolus into the nucleoplasm; something that also happens during infection by *P. infestans*. Pi04314 interaction is mediated by an R/KVxF motif, indicating that Pi04314 mimics PP1c regulatory subunits. Either VIGS of the *PP1c* isoforms, or overexpression of a phosphatase-dead mutant of PP1c, reduces *P. infestans* infection, indicating that PP1c is required for infection. Indeed, the effector does not inhibit PP1c phosphatase activity, but rather is predicted to form a holoenzyme with PP1c, potentially directing its activity to the dephosphorylation of substrates involved in plant defence [55^••^]. In addition to being defined as an S factor, PP1c can also be considered an ‘effector helper’; a host protein that is co-opted by the effector to help modify defence proteins [53, 57]. The RXLR effector Pi02860 interacts in the cytoplasm and at the host PM with a non-phototrophic hypocotyl 3/Root phototropism 2 (NPH3/RPT2)-Like protein, NRL1 (Figure 1), which is predicted to form a ubiquitin E3 ligase with Cullin 3 [56^•^]. Remarkably, over-expression of NRL1 suppresses INF1-triggered cell death and enhances *P. infestans* leaf colonisation, whereas silencing of *NRL1* by VIGS accelerates INF1-triggered cell death and reduces pathogen colonisation. NRL1 is a negative regulator of immunity, and thus a further S factor targeted by a *P. infestans* RXLR effector [56^•^]. Given that plants have well-tuned endogenous mechanisms for negative regulation of immunity, it is not surprising that pathogens have evolved effectors to exploit this.

## Conclusions and future work

The cell biology of late blight disease has focussed heavily in recent years on the activities of effectors. Great strides have been made in identifying the targets of RXLR effectors and in characterising the changes these effectors make inside the plant cell to promote disease. The haustorium is recognised as a site for delivery of RXLR effectors. Indeed, growing evidence suggests the haustorium may be a general site of secretion, both conventional and non-conventional, during infection. Nevertheless, the precise means by which RXLR or CRN effectors may be translocated into plant cells remains unknown. Protein secretion from *P. infestans* during infection needs to be studied systematically to determine the spatiotemporal dynamics of cytoskeletal proteins such as actin and tubulin, transport proteins such as dynamin, kinesin and myosin, and protein complexes such as the exocyst, with a role in exocytosis. Alternative pathways for protein secretion are also an emerging area for investigation in oomycetes. In mammalian pathosystems, non-conventional protein secretion has been found to involve exosomes, tiny membrane vesicles produced from pathogen cells that can fuse with host cells to deliver virulence proteins (reviewed in [58]). Future cell biological studies could determine the potential involvement of exosomes in effector delivery by *P. infestans*. In addition, important observations have highlighted the unique nature of the EHM, and the potential role for plant endocytic processes in its biogenesis. The precise mechanisms behind EHM biogenesis warrant further detailed study, as does the contribution that pathogen effectors make to shaping and controlling this biogenic process.

The general cell biology of *P. infestans* and other oomycetes is relatively poorly studied compared to model fungi such as *Neuropspora crassa*, or fungal pathogens such as *Magnaporthe oryzae* and *Ustilago maydis.* An important benefit that could arise from expanding such studies would be the development of new agrochemical control compounds. By combining a detailed knowledge of the molecular processes required for disease, with improved cell biology resources and tools, it may be possible to identify new targets for chemical control, or new chemical classes with activity against validated targets. Specific pathogen cellular components may be labelled, and phenotyping assays developed to screen compounds for the ability to disrupt these processes and inhibit pathogen growth or infection. These assays can be used in high-throughput confocal microscopy to quantify changes caused by exposure of *Phytophthora* to tested chemicals. Such high-content screening strategies are being used for discovery of new drugs to combat human diseases [59, 60, 61, 62] and could help to revolutionise the generation of new mechanisms of action for chemical control of this economically devastating crop disease.

## References and recommended reading

Papers of particular interest, published within the period of review, have been highlighted as:

• of special interest
•• of outstanding interest
